# A case of adult undifferentiated embryonal sarcoma of the liver successfully treated with right trisectionectomy: a case report

**DOI:** 10.1186/s40792-017-0295-1

**Published:** 2017-01-31

**Authors:** Akiko Mori, Koji Fukase, Kunihiro Masuda, Naoaki Sakata, Masamichi Mizuma, Hideo Ohtsuka, Takanori Morikawa, Kei Nakagawa, Hiroki Hayashi, Fuyuhiko Motoi, Takeshi Naitoh, Keigo Murakami, Michiaki Unno

**Affiliations:** 1 Department of Surgery, Tohoku University, 1-1 Seiryo-machi, Aoba-ku, Sendai, Miyagi 980-8574 Japan; 2 Department of Pathology, Tohoku University, 1-1 Seiryo-machi, Aoba-ku, Sendai, Miyagi 980-8574 Japan

**Keywords:** Undifferentiated embryonal sarcoma of the liver, Adult, Mesenchymal hamartoma of the liver

## Abstract

**Background:**

Undifferentiated embryonal sarcoma of the liver (UESL) is a rare malignant mesenchymal tumor that usually occurs in children and is rarely diagnosed in adults.

**Case presentation:**

Here, we describe the case of a 65-year-old woman who presented with a huge cystic lesion in the liver. Laboratory studies performed on admission showed modest inflammation, poor nutrition, and elevated levels of total bilirubin, alkaline phosphatase, and γ-glutamyl transferase. Computed tomography showed a well-defined, heterogeneous tumor with multiple cysts involving the right lobe and the medial segment of the liver, with a maximum diameter of 16 cm. Positron emission tomography/computed tomographic scans showed the uptake of 2-(fluorine-18)-fluoro-2-deoxy-d-glucose in a part of the cyst. The patient was diagnosed with mucinous cystadenocarcinoma or sarcoma of the liver and underwent right trisectionectomy. Histopathological studies revealed that the tumor was composed of pleomorphic and polynuclear dyskaryotic cells with eosinophilic globules in the cytoplasm. Mesenchymal hamartoma-like tissue was observed in the peripheral part of the tumor. Immunohistochemical analyses showed the tumor stained with vimentin, α-smooth muscle actin, desmin, α1-antitrypsin, and α1-antichymotripsin. Therefore, a histological diagnosis of UESL was made. Eighteen months following treatment, two recurrent tumors in the remnant liver were detected and resection of the recurrent tumors was performed.

**Conclusions:**

A UESL should be considered in the differential diagnosis of large cystic hepatic lesions. Although the prognosis of UESL is extremely unfavorable, aggressive surgical resection should be the most important factor for ensuring long-term survival.

## Background

Undifferentiated embryonal sarcoma of the liver (UESL) is a rare and aggressive malignant tumor that is observed predominantly in children [1]. To the best of our knowledge, fewer than 40 cases of UESL have been reported in patients older than 20 years over the past 50 years [2]. Therefore, it is important to consider this entity in the differential diagnosis of a large hepatic mass. The pathological features of UESL are poorly understood. A link between UESL and mesenchymal hamartoma of the liver (MHL) has been proposed because of the overlapping clinicopathological features of these entities [3]. In this report, we describe a case of UESL in an adult with historical features of an MHL-like lesion that had a good prognosis with complete resection.

## Case presentation

**Fig. 1:**
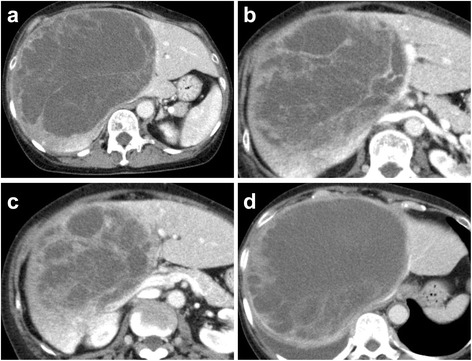
Abdominal CT revealed a huge tumor. A well-defined, low-density, heterogeneous, multilocular, cystic tumor with septa involved the right lobe and the medial segment of the liver (**a**). The tumor compressed the umbilical portion of the portal vein (**b**), the right portal vein (**c**), and the left hepatic vein (**d**)

**Fig. 2:**
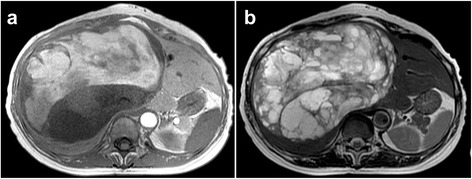
MRI revealed high and low signal intensity in T1-weighted images (**a**) and high signal intensity in T2-weighted images (**b**)

**Fig. 3:**
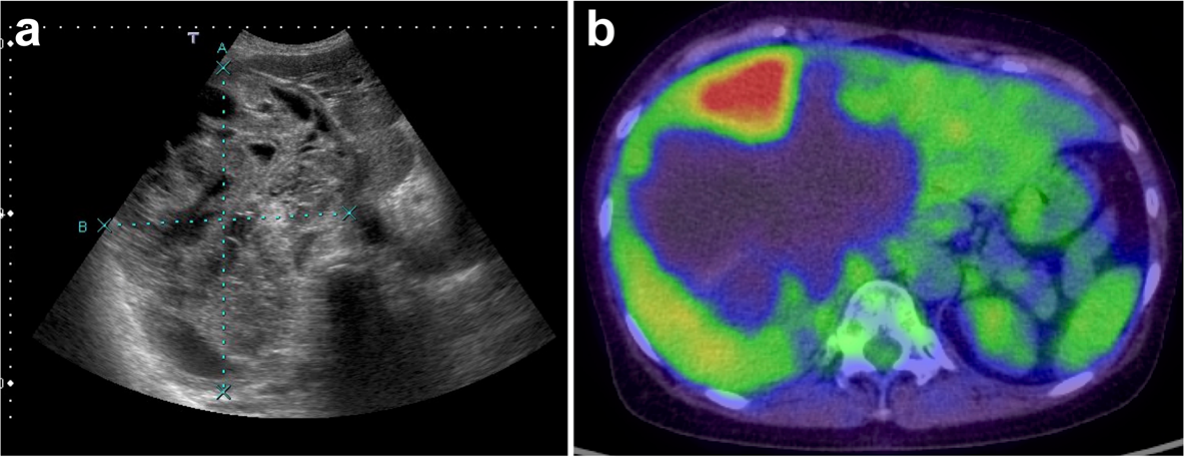
Abdominal US revealed a honeycomb pattern in the tumor, with solid components (**a**). PET-CT showed the uptake of FDG in the solid components of the cyst (**b**)

**Fig. 4:**
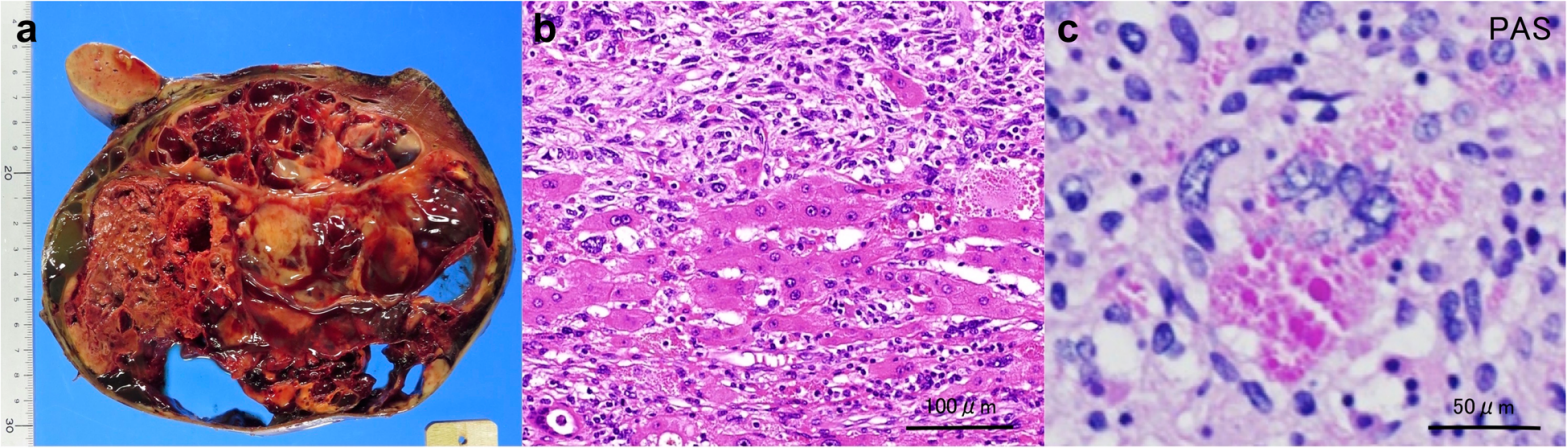
Pathological findings of the tumor. Macroscopically, the cut surface revealed a multilocular mass with areas of hemorrhage, necrosis, and a mucinous substance (**a**). Microscopically, the tumor was composed of pleomorphic and polynuclear dyskaryotic cells, and mesenchymal hamartoma-like lesions were partially seen (hematoxylin and eosin stain) (**b**). The cytoplasm of the polynuclear dyskaryotic cell contained eosinophilic globules that were diastase-resistant PAS positive (PAS stain) (**c**)

**Fig. 5:**
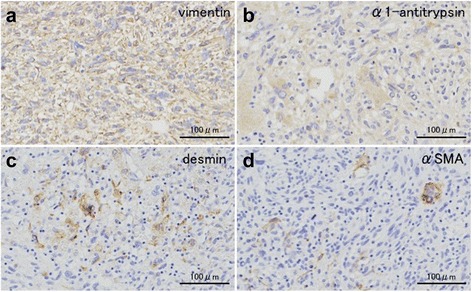
Immunohistochemical analysis showed that the tumor was stained with vimentin (**a**), α1-antitrypsin (**b**), desmin (**c**), and α-smooth muscle actin (**d**)

**Fig. 6:**
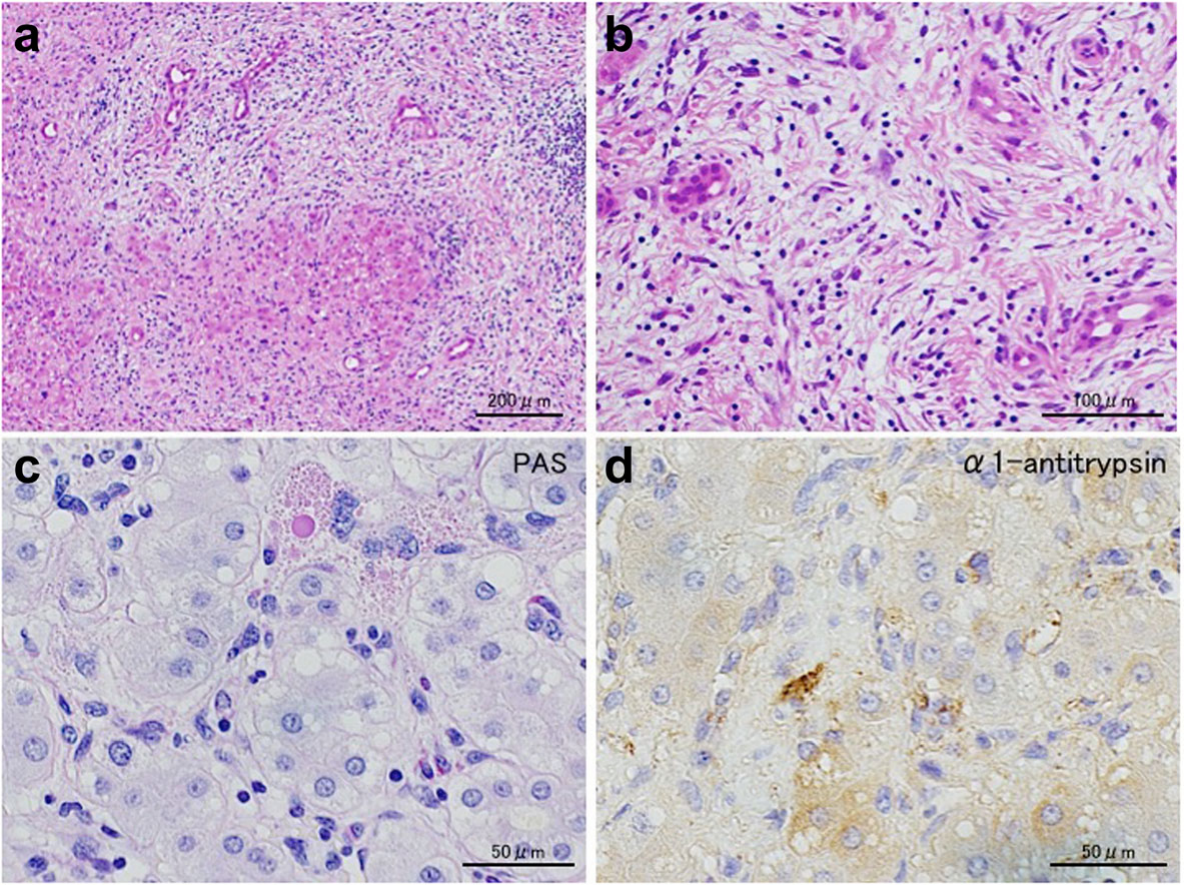
Pathological findings of the metastatic tumor. The tumor was composed of pleomorphic and polynuclear dyskaryotic cells (hematoxylin and eosin stain) (**a**). Mesenchymal hamartoma-like lesions were partially seen (hematoxylin and eosin stain) (**b**). The cytoplasm of the polynuclear dyskaryotic cell contained eosinophilic globules that were diastase-resistant PAS positive (PAS stain) (**c**). Immunohistochemically, the tumor was positive for α1-antitrypsin (**d**)

A 65-year-old woman presented to our hospital for further examination of a large hepatic tumor that has been detected on abdominal computed tomography (CT) performed at another hospital to evaluate right dorsal pain. Laboratory studies showed slightly increased values of hepatobiliary enzymes, including total bilirubin (1.3 mg/dL), alkaline phosphatase (452 IU/L), and γ-glutamyl transferase (167 IU/L). The indocyanine green plasma disappearance rate (0.103) and 15-min retention rate (23.9%) suggested depressed liver function. Tests for serum hepatitis B and C viral markers were negative. The levels of α-fetoprotein (AFP), protein induced by vitamin K absence-II (PIVKA-II), carcinoembryonic antigen (CEA), and carbohydrate antigen 19-9 (CA19-9) were all within normal limits, whereas elevated values were observed for Duke pancreatic monoclonal antigen type 2 (DUPAN-2; 200 U/mL), cancer antigen 125 (CA125; 321.8 U/mL), and neuron specific enolase (NSE; 29.2 ng/mL). Abdominal CT revealed a well-defined, low-density, heterogeneous, multilocular, cystic tumor with septa replacing the right hepatic lobe and the medial segment of the left hepatic lobe, measuring 16 cm in maximum diameter. The large tumor contained an enhancing solid compartment and a non-enhancing cystic compartment. It compressed the umbilical portion of the portal vein, the right portal vein, and the left hepatic vein (Fig. 1). Magnetic resonance imaging (MRI) showed a mixed intensity of high and low signals. The cysts had low signal intensity on T1-weighted images and had high signal intensity on T2-weighted images, suggesting high water content. Furthermore, areas with partially high signal intensity on T1-weighted images and low signal intensity on T2-weighted images were suggestive of intratumoral hemorrhage (Fig. 2). Abdominal ultrasonography (US) showed a honeycomb-like tumor with solid components (Fig. 3a). Positron emission tomography (PET) showed the uptake of 2-(fluorine-18)-fluoro-2-deoxy-d-glucose (FDG) in part of the solid components of the cyst (Fig. 3b). From these imaging findings, the preoperative diagnosis of mucinous cystadenocarcinoma or sarcoma of the liver was made. As the right hepatic lobe was occupied by the large tumor and the percentage of the future remnant liver volume in right hepatic trisectionectomy was 52.3%, we did not perform preoperative portal vein embolization. After nutritional therapy, the indocyanine green plasma disappearance rate (0.150) and 15-min retention rate (11.9%) were recovered. We decided to perform right trisectionectomy with caudate lobectomy. An inverted T-shaped incision with thoracotomy along the ninth intercostal space was performed. Intraoperative findings revealed that the large tumor was well circumscribed and showed no invasion of vessels, so bile duct resection and vascular resection were not performed. The right hepatic artery and the right portal vein were divided and ligated. After mobilization of the right liver, a number of short hepatic veins were also ligated and divided. The right and middle hepatic veins were dissected and ligated with an Endo GIA. We then divided the liver parenchyma with a Pringle maneuver. Because lymph node swelling was not observed, a regional lymph node dissection was not performed. Operation time was 364 min and total blood loss was 2521 ml. The resected specimen showed a heterogenic tumor measuring 19 × 17 × 13 cm. The cut surface of the specimen revealed a multilocular mass with the various components of hemorrhage, necrosis, and a mucinous substance. Microscopically, the tumor was composed of pleomorphic and polynuclear dyskaryotic cells with eosinophilic globules in its cytoplasm, which were diastase-resistant Periodic acid–Schiff (PAS) positive. The area of FDG uptake in PET was mainly consistent with viable tumor cells. In the peripheral part of the area, mesenchymal hamartoma-like lesions consisting of loose myxoid stroma and non-atypical spindle cells were observed (Fig. 4). No vascular invasion or intrahepatic metastases were detected. The surgical margin was free (R0). According to immunohistochemistry, the tumor was positive for vimentin, α1-antitrypsin, and α1-antichymotripsin. Some tumor cells also expressed desmin and α-smooth muscle actin (Fig. 5). On the basis of these findings, the tumor was histologically diagnosed as UESL. After surgery, the patient did not receive adjuvant chemotherapy and has been followed with imaging studies, including a whole-body CT and PET scan. Eighteen months following treatment, recurrent tumors in the remnant liver were detected in S3 and partial resection of the liver was performed. Microscopically, the tumors consisted of atypical cells with irregular nuclei and polynuclear dyskaryotic cells with eosinophilic globules in the cytoplasm, which were diastase-resistant PAS positive (Fig. 6a–c). By immunohistochemistry, these atypical cells stained with α1-antitrypsin (Fig. 6d). Findings indicated recurrent UESL. Moreover, a part of the tumor consisted of a loose edematous matrix populated by spindle or stellate-shaped cells with bile ducts and small cystic spaces, which was reminiscent of a mesenchymal hamartoma. The patient is currently alive 26 months following the first operation and has no recurrent tumor.

## Discussion

UESL is a relatively new clinicopathologic entity that was first described in 1978 by Stocker and Ishak [4]. It is found most frequently in children between 6 and 10 years of age [4] but has been rarely reported in adults. There have been 46 articles regarding adult UESL published from 1977 to 2015 [5]. UESL accounts for fewer than 1% of all primary liver neoplasms in adults [1]. In a recent review on UESL, the median age of the study group was 25, with the oldest patient being 86 years old [6]. In most cases, symptoms related to the tumor are nonspecific, with abdominal pain and abdominal mass reported to be the most common presenting complaints [2, 7, 8]. There are no specific tumor markers. Furthermore, the results of imaging studies such as CT, US, and MRI are often inconclusive [1]. On US, UESL usually appears as a hypoechoic solid mass [9]. CT characteristics include the presence of an enhanced peripheral rim, some solid portions at the periphery or adjacent to the septa, and a predominantly unilocular or multilocular appearance [9, 10]. These features occasionally lead to this tumor being misdiagnosed as a benign cystic lesion. In this case, because of its multilocular cystic appearance on CT imaging, mucinous cystadenocarcinoma of the liver should be considered in the differential diagnosis. In addition, since UESL in adulthood is very rare, it was extremely difficult in this case to make a preoperative diagnosis. However, the intermixed appearance of hemorrhage and mucinous content on MRI and the honeycomb appearance with solid components on US studies were the characteristics of UESL. The discrepancy between the solid appearance on US and cystic appearance on CT in particular should raise suspicion for this tumor [9].

The prognosis of UESL is very poor, with a median reported survival time of less than 1 year [4]. Some case reports and studies demonstrated long-term survival of patients who underwent only surgery [11]. However, even if a complete resection is achieved in surgery, UESL recurs in many cases [6]. Lenze et al. reviewed reports from 1955 to 2007 and reported that the combination of surgical resection and adjuvant chemotherapy may improve prognosis compared with radical tumor resection alone [6]. Although there were no standard regimens for adjuvant chemotherapy, a sarcoma-directed chemotherapy such as combination of vincristine, actinomycin, ifosfamide, and doxorubicin or combination of carboplatin and etoposide was used [1, 6]. In addition, multidisciplinary therapy such as chemotherapy, radiotherapy, interventional therapy, and transplantation may be helpful for improving survival for unresectable recurrent tumors [12]. However, adjuvant chemotherapy was not administered to our patient because there were no standard regimens for UESL. Repeat complete resection for recurrent tumor was performed, and now, the patient has no recurrent tumor after 26 months following the first operation.

The pathological features of UESL are poorly understood. In the case reported here, mesenchymal hamartoma-like lesions were partially observed. Several authors have suggested an evolutive histological spectrum between MHL and UESL, based on histological findings [13, 14]. However, the occurrence of MHL in adults is also very rare. Furthermore, there are some reports of a benign postoperative course of MHL after incomplete resection [15–19]. In the liver metastatic lesion of this case, there were some mesenchymal hamartoma-like lesions. A UESL-like lesion and a mesenchymal hamartoma-like lesion coexisted not only in the primary tumor but also in the metastatic tumor, suggesting that a mixed mesenchymal hamartoma-like lesion is one form of UESL, rather than a malignant transformation of MHL. Further investigation is needed to clarify the pathogenesis and relationship of MHL and UESL.

## Conclusions

It is important to achieve accurate and early diagnosis of UESL and include it in the differential diagnosis of large liver masses, regardless of the patient’s age. Although UESL is an aggressive tumor with a poor prognosis, the patient reported herein experienced a good survival through aggressive liver resection, including repeat hepatectomy. Therefore, complete resection should be the most important factor for ensuring long-term survival.

## Abbreviations

CT: Computed tomography
FDG: 2-(Fluorine-18)-fluoro-2-deoxy-d-glucose
MHL: Mesenchymal hamartoma of the liver
MRI: Magnetic resonance imaging
PAS: Periodic acid–Schiff
PET: Positron emission tomography
UESL: Undifferentiated embryonal sarcoma of the liver
US: Ultrasonography
